# Effective protection against acute respiratory distress syndrome/sepsis injury by combined adipose-derived mesenchymal stem cells and preactivated disaggregated platelets

**DOI:** 10.18632/oncotarget.19312

**Published:** 2017-07-17

**Authors:** Chih-Hung Chen, Yung-Lung Chen, Pei-Hsun Sung, Cheuk-Kwan Sun, Kuan-Hung Chen, Yi-Ling Chen, Tien-Hung Huang, Hung-I Lu, Fan-Yen Lee, Jiunn-Jye Sheu, Sheng-Ying Chung, Mel S. Lee, Hon-Kan Yip

**Affiliations:** ^1^ Divisions of General Medicine, Department of Internal Medicine, Kaohsiung Chang Gung Memorial Hospital and Chang Gung University College of Medicine, Kaohsiung, Taiwan; ^2^ Division of Cardiology, Department of Internal Medicine, Kaohsiung Chang Gung Memorial Hospital and Chang Gung University College of Medicine, Kaohsiung, Taiwan; ^3^ Department of Emergency Medicine, E-Da Hospital, I-Shou University School of Medicine for International Students, Kaohsiung, Taiwan; ^4^ Department of Anesthesiology, Kaohsiung Chang Gung Memorial Hospital and Chang Gung University College of Medicine, Kaohsiung, Taiwan; ^5^ Division of Thoracic and Cardiovascular Surgery, Department of Surgery, Kaohsiung Chang Gung Memorial Hospital and Chang Gung University College of Medicine, Kaohsiung, Taiwan; ^6^ Department of Orthopedics, Kaohsiung Chang Gung Memorial Hospital and Chang Gung University College of Medicine, Kaohsiung, Taiwan; ^7^ Institute for Translational Research in Biomedicine, Kaohsiung Chang Gung Memorial Hospital, Kaohsiung, Taiwan; ^8^ Center for Shockwave Medicine and Tissue Engineering, Kaohsiung Chang Gung Memorial Hospital, Kaohsiung, Taiwan; ^9^ Department of Medical Research, China Medical University Hospital, China Medical University, Taichung, Taiwan; ^10^ Department of Nursing, Asia University, Taichung, Taiwan

**Keywords:** acute respiratory distress syndrome, sepsis, inflammation, adipose-derived mesenchymal stem cell, platelets

## Abstract

This study assessed whether combining adipose-derived mesenchymal stem cells (ADMSC) with preactivated, disaggregated shape-changed platelets (PreD-SCP) was superior to either therapy alone for protecting rat lung from acute respiratory distress syndrome (ARDS) complicated by sepsis. ARDS and sepsis were induced through 100% oxygen inhalation and peritoneal administration of 1.5 mg/kg lipopolysaccharide (LPS), respectively. Adult-male Sprague-Dawley rats (n=40) were randomized into sham-control (SC), ARDS-LPS, ARDS-LPS-ADMSC (1.2x10^6^ cells), ARDS-LPS-PreD-SCP (3.0x10^8^, intravenous administration), and ARDS-LPS-ADMS/PreD-SCP groups, and were sacrificed 72 h after 48 h ARDS induction. Lung injury scores (LIS) and collagen deposition were highest in ARDS-LPS, lowest in SC, higher in ARDS-LPS+ADMSC than in ARDS-LPS+PreD-SCP and ARDS-LPS+ADMS/PreD-SCP, and higher in ARDS-LPS+PreD-SCP than in ARDS-LPS+ADMS/PreD-SCP (all p<0.0001). Alveolar-sac numbers, oxygen saturation, endothelial marker levels, and mitochondrial cytochrome-C levels exhibited opposite patterns with respect to LIS (all p<0.001). Levels of inflammatory, oxidative-stress, apoptosis, mitochondrial/DNA damage, and MAPK and Akt signaling markers exhibited patterns identical to that of LIS (all p<0.001). Anti-oxidant and anti-inflammatory protein levels increased progressively from SC to ARDS-LPS+ADMS/PreD-SCP (all p<0.0001). These findings indicate combined ADMSC/PreD-SCP was superior to either therapy alone for protecting rat lung from ARDS-sepsis injury.

## INTRODUCTION

The lung is particularly vulnerable to acute injury in various situations, including cardiopulmonary bypass, resuscitation following cardiac arrest, hypoxic respiratory failure, smoke or toxic chemical inhalation, respiratory failure requiring high concentration oxygen supply and prolonged mechanical ventilator support, viral infection, and sepsis/septic shock [1–13]. These insults can lead to the development of acute respiratory distress syndrome (ARDS), the most life-threatening type of acute lung injury (ALI) [3, 12].

Despite state-of-the art therapeutic and intensive care strategies [1, 2, 8, 10, 11], ARDS is not only a leading cause of morbidity and mortality in hospitalized patients, but also has an unacceptably poor long-term outcome, especially in severe cases [14–16]. Innovative, safe, and effective therapeutic options are needed to improve short- and long-term patient outcomes. However, a thorough understanding of underlying ARDS mechanisms is a pre-requisite for possible breakthroughs. ALI and ARDS mechanisms are likely multifactorial and include inflammation, alveolar leukocytosis, leaked proteins, reactive oxygen species (ROS) generation, and apoptosis [5, 12, 17–21].

Cell-based therapy offers a new therapeutic option for ischemia-related organ dysfunctions refractory to traditional treatments. Stem cells, especially adipose-derived mesenchymal stem cells (ADMSCs), can ameliorate inflammation and oxidative stress [20, 22], and stem cell therapy exhibits immunomodulatory capabilities [20, 22, 23]. Similarly, platelet-rich plasma (PRP) is anti-inflammatory due to its ability to suppress production of tumor necrosis factor (TNF)-α and interleukin (IL)-1β, two principal pro-inflammatory cytokines [24, 25]. Our recent study showed that preactivated and disaggregated shape-changed platelets (PreD-SCP) treatment alleviated lung injury in ARDS complicated by sepsis via their anti-inflammatory and anti-oxidative activities [26].

Combination therapy is a very common strategy for treating various diseases, such as hypertension, heart failure, diabetes mellitus, and HIV/AIDS. Combination therapy allows for drug synergy that can result in reduced therapeutic doses and toxicities. We proposed that combined ADMSC and PreD-SCP therapy might have an additive effect in protecting lung structural and functional integrity against the impacts of ARDS complicated by sepsis injury. The present study investigated the therapeutic potential of early combined ADMSC and PreD-SCP treatment in a rat model of ARDS complicated by sepsis.

## RESULTS

Results are described with respect to tissue samples collected 5 d (i.e., included 48 h ARDS induction) after the initiation of ARDS and sepsis induction in rats.

### Arterial oxygen saturation (SaO_2_), total wet lung weight, and lung injury score

**Figure 1:**
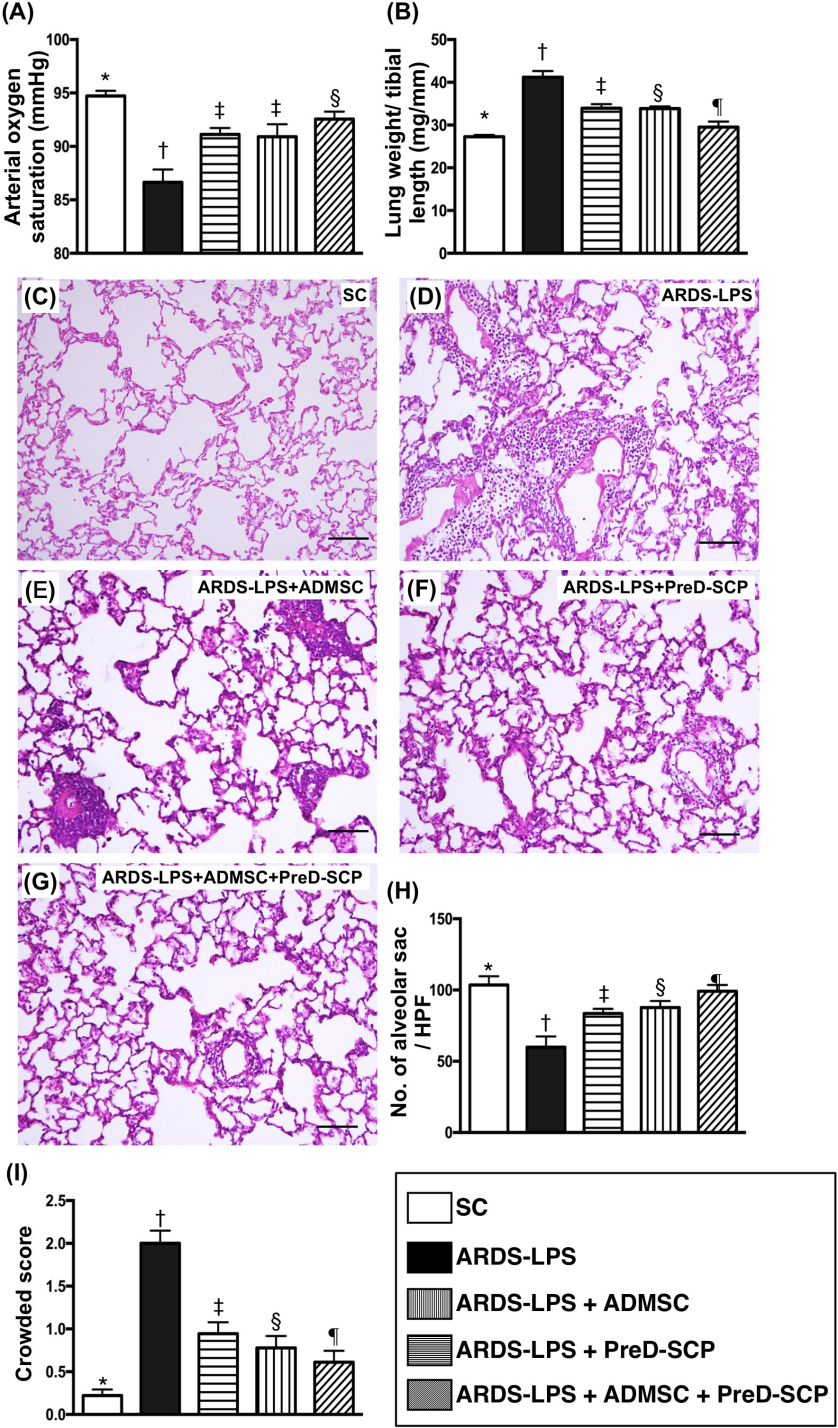
Arterial oxygen saturation (SaO_2_), wet lung weight and lung injury score 5 d after ARDS and sepsis induction SaO_2_ (%) **(A)** and total wet lung weight **(B)** * vs. other groups with different symbols (†, ‡, §), p<0.0001. H&E staining to assess numbers of alveolar sacs and lung crowd score (100x) **(C–G)**. Scale bars in right lower corner represent 100μm. Number of alveolar sacs **(H)** * vs. other groups with different symbols (†, ‡, §, ¶), p<0.0001. Lung parenchymal crowded score **(I)** * vs. other groups with different symbols (†, ‡, §, ¶), p<0.0001. N=8 for each group. (*, †, ‡, §, ¶) p<0.05. SC, sham control; ARDS-LPS, acute respiratory distress syndrome-lipopolysaccharide; PreD-SCP, preactivated and disaggregated shape-changed platelet; ADMSC, adipose-derived mesenchymal stem cell.

SaO_2_ was highest in group 1 (SC) and lowest in group 2 (ARDS-LPS). SaO_2_ was lower in groups 3 (ARDS-LPS + ADMSC) and 4 (ARDS-LPS + PreD-SCP) as compared to group 5 (ARDS-LPS + ADMSC + PreD-SCP), but there was no difference between groups 3 and 4 (Figure 1A). Total wet lung weight exhibited the opposite pattern compared to SaO_2_ among the five groups (Figure 1B), suggesting that fluid and protein in the lung parenchyma following capillary leakage lead to ARDS-sepsis damage.

H&E tissue staining analyses of rats from all five groups (Figure 1C–1G) demonstrated the following pattern with respect to numbers of alveolar sacs: group 1>5>4>3>2 (Figure 1H). Lung parenchymal crowding showed the opposite pattern (Figure 1I).

### Sirius red and α-SMA staining for collagen deposition and small vessels in lung parenchyma

**Figure 2:**
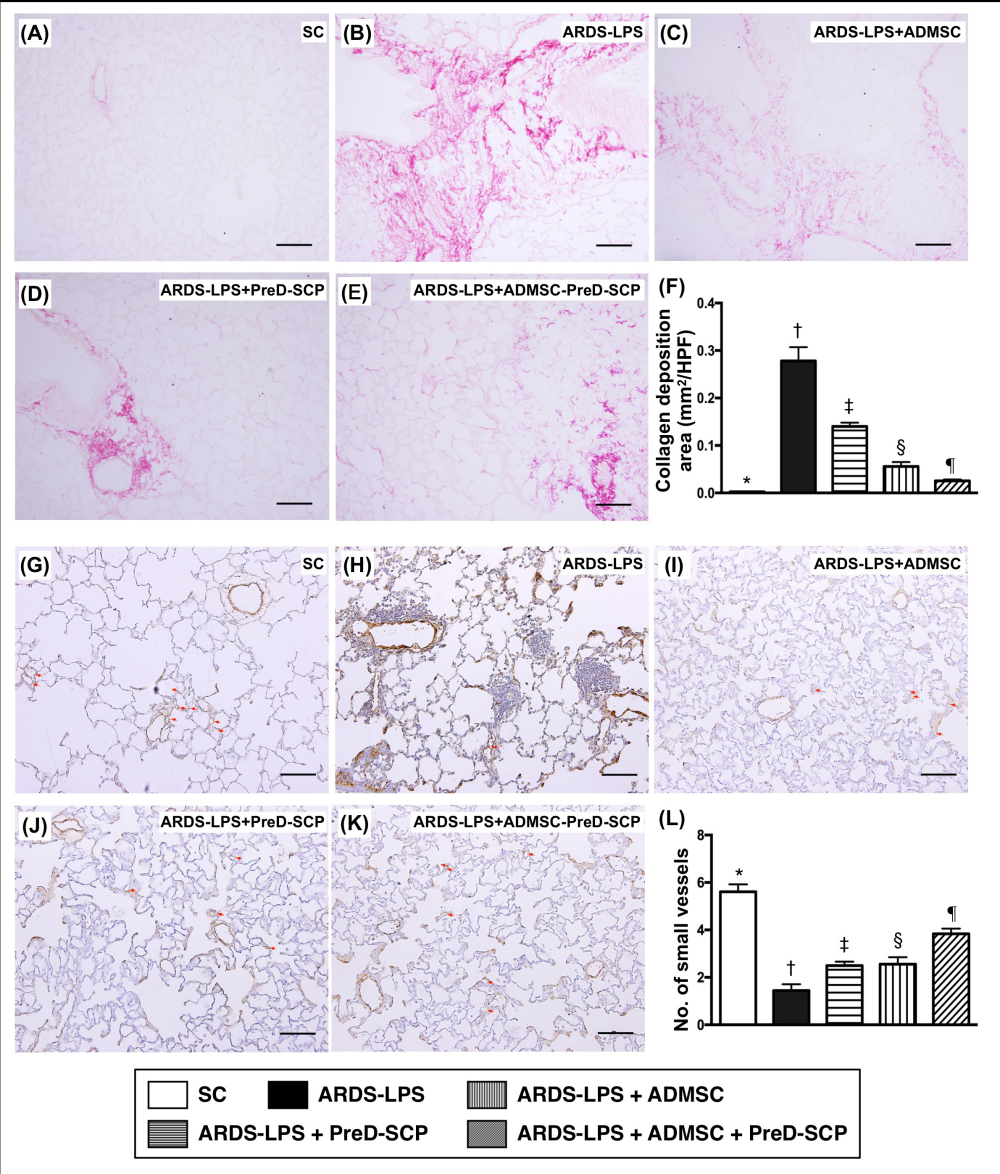
Collagen deposition and numbers of small vessels in lung parenchyma 5 d after ARDS and sepsis induction Sirius red staining for collagen deposition (pink color) in lung parenchyma (100x) **(A–E)**. Collagen deposition **(F)** * vs. other groups with different symbols (†, ‡, §, ¶), p<0.0001. α-smooth muscle actin (SMA) staining for small vessels (gray color; diameter <25 μM) (100x) **(G–K).** Numbers of small vessels **(L)** * vs. other groups with different symbols (†, ‡, §, ¶), p<0.0001. Scale bars in right lower corner represent 100μm. N=8 for each group. (*, †, ‡, §, ¶) p<0.05. SC, sham control; ARDS-LPS, acute respiratory distress syndrome-lipopolysaccharide; PreD-SCP, preactivated and disaggregated shape-changed platelet; ADMSC, adipose-derived mesenchymal stem cell; HPF, high-power field.

Collagen deposition in lung parenchyma as identified by Sirius red staining showed the following pattern in terms of scores: group 2>3>4>5>1. (Figure 2A–2F). α-smooth muscle actin (α-SMA) staining for quantification of small vessels (<25 μm) exhibited the opposite pattern (Figure 2L).

### Inflammatory, apoptotic, and anti-apoptotic biomarkers in lung parenchyma

**Figure 3:**
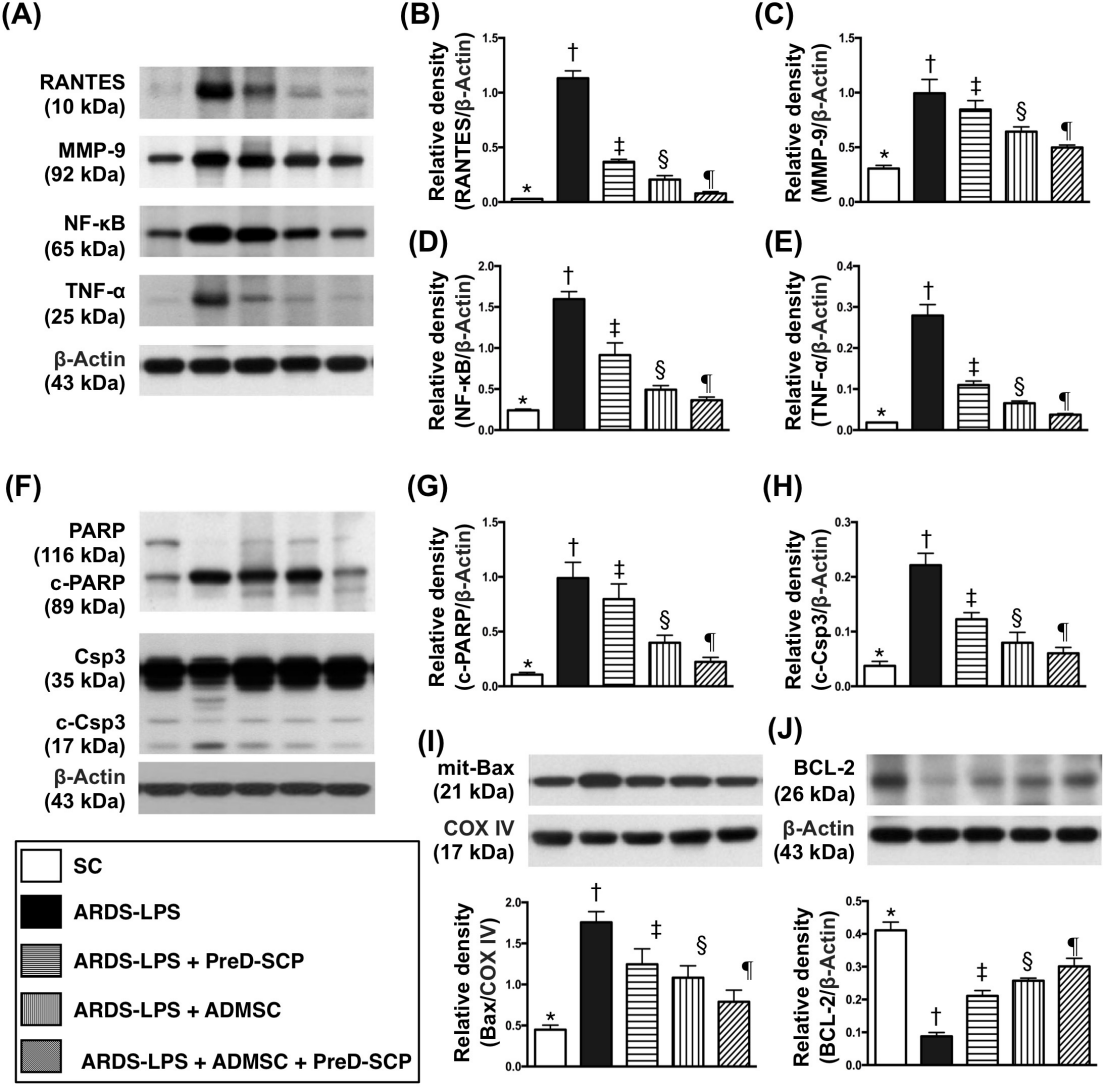
Inflammatory, apoptotic, and anti-apoptotic biomarkers in lung parenchyma 5 d after ARDS and sepsis induction Protein levels with different ß-actin as internal controls **(A) & (F)**. Levels of RANTES **(B)**, MMP-9 **(C)**, NF-κB **(D)**, TNF-α **(E)**, cleaved PARP (c-PARP) **(G)**, cleaved caspase (c-Csp) 3 **(H)**, mitochondrial (mit) Bax **(I)**, Bcl-2 **(J)** * vs. other groups with different symbols (†, ‡, §, ¶), p<0.0001. N=8 for each group. (*, †, ‡, §, ¶) p<0.05 level. SC, sham control; ARDS-LPS, acute respiratory distress syndrome-lipopolysaccharide; PreD-SCP, preactivated and disaggregated shape-changed platelet; ADMSC, adipose-derived mesenchymal stem cell.

Matrix metalloproteinase (MMP)-9, tumor necrosis factor (TNF)-α, nuclear factor (NF)-κB and RANTES, four inflammation indicators, along with three apoptosis indices, mitochondrial Bax, cleaved caspase 3, and cleaved poly (ADP-ribose) polymerase (PARP), exhibited the following pattern with respect to measured protein levels: group 2>3>4>5>1 (Figure 3). Bcl-2, an anti-apoptosis marker, exhibited the opposite pattern.

### Mitochondrial and DNA damage biomarkers and oxidative stress in lung parenchyma

**Figure 4:**
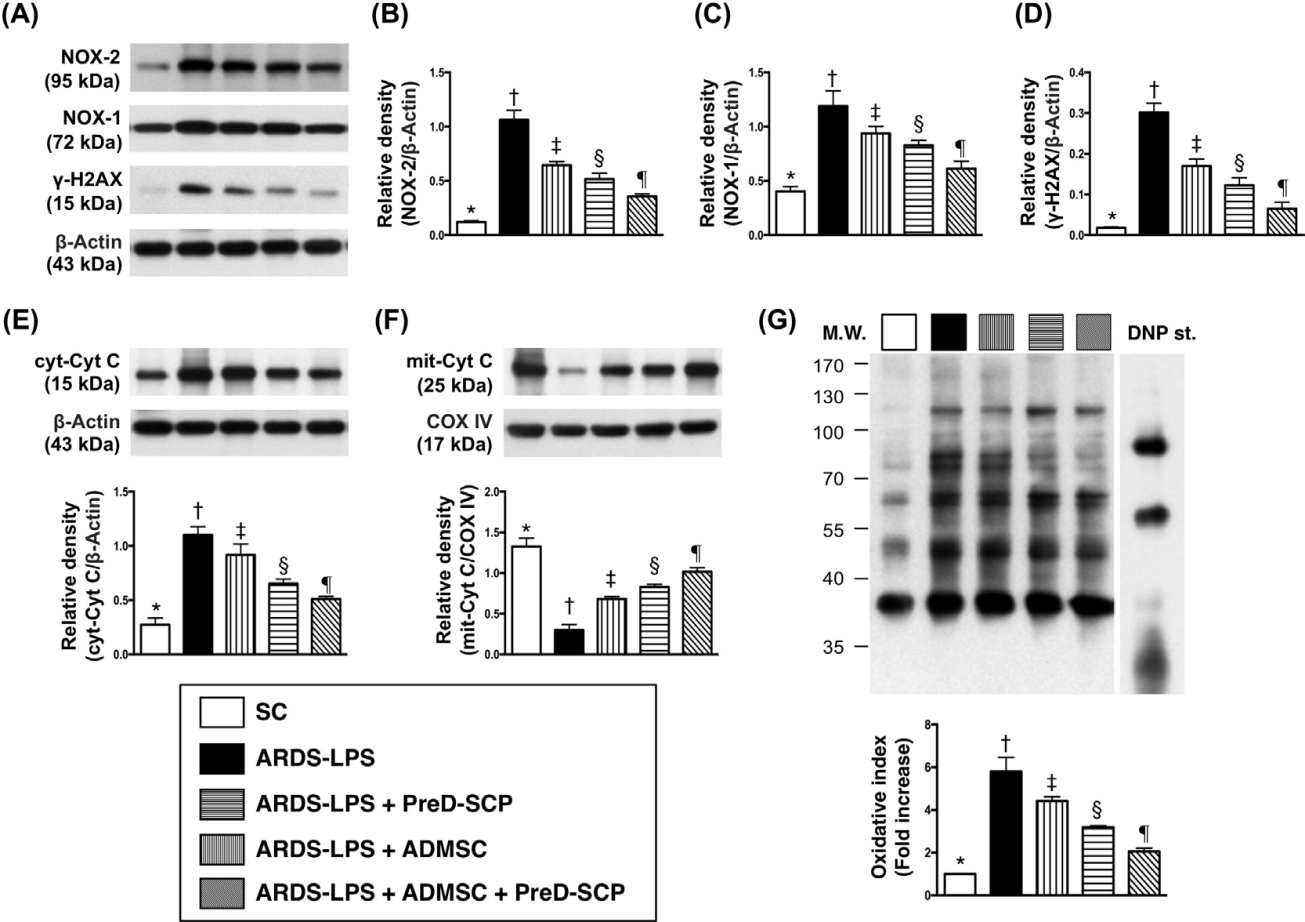
Mitochondrial and DNA damage biomarkers, and oxidative stress in lung parenchyma 5 d after ARDS and sepsis induction NOX-1, NOX2, and γ-H2AX with ß-actin as the internal control **(A).** NOX-2 **(B)**, NOX-1 **(C)**, γ-H2AX **(D)**, cytosolic cytochrome C (c-Cyto c) **(E)**, mitochondrial cytochrome C (m-Cyto c) **(F)** * vs. other groups with different symbols (†, ‡, §, ¶), p<0.0001. Oxidized protein expression **(G)** * vs. other groups with different symbols (†, ‡, §), p<0.0001. Upper panel: left and right lanes represent MW marker and control oxidized molecular protein standard, respectively. N=8 for each group. (*, †, ‡, §, ¶) p<0.05. SC, sham control; ARDS-LPS, acute respiratory distress syndrome-lipopolysaccharide; PreD-SCP, preactivated and disaggregated shape-changed platelet; ADMSC, adipose-derived mesenchymal stem cell; MW, molecular weight; DNP, 1-3 dinitrophenylhydrazone.

γ-H2AX, a DNA damage indicator, cytosolic cytochrome C, a mitochondrial damage indicator, and NOX-1, NOX-2, and oxidized protein in lung tissue, three indicators of oxidative stress, exhibited the following pattern with respect to measured protein levels: group 2>3>4>5>1 (Figure 4). Mitochondrial cytochrome C, an index of mitochondrial integrity, showed the opposite pattern.

### Anti-inflammatory biomarkers, and MAPK family and Akt signaling pathways

**Figure 5:**
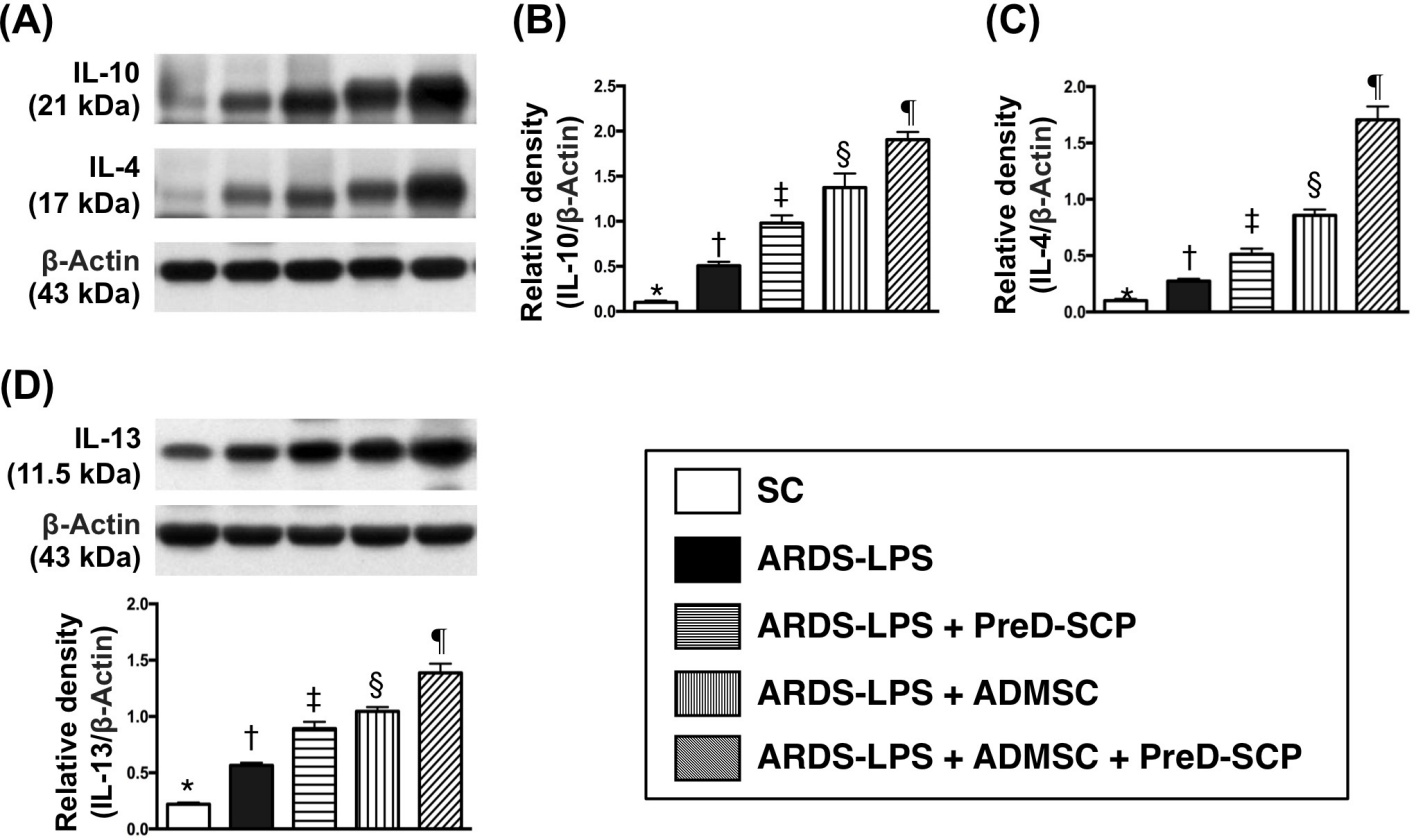
Anti-inflammatory biomarkers 5 d after ARDS and sepsis induction IL-10 and IL-4 with ß-actin as the internal control **(A)**. IL-10 **(B)**, IL-4 **(C)**, IL-13 **(D)** * vs. other groups with different symbols (†, ‡, §, ¶), p<0.0001. N=8 for each group. (*, †, ‡, §, ¶) p<0.05. SC, sham control; ARDS-LPS, acute respiratory distress syndrome-lipopolysaccharide; PreD-SCP, preactivated and disaggregated shape-changed platelet; ADMSC, adipose-derived mesenchymal stem cell.

**Figure 6:**
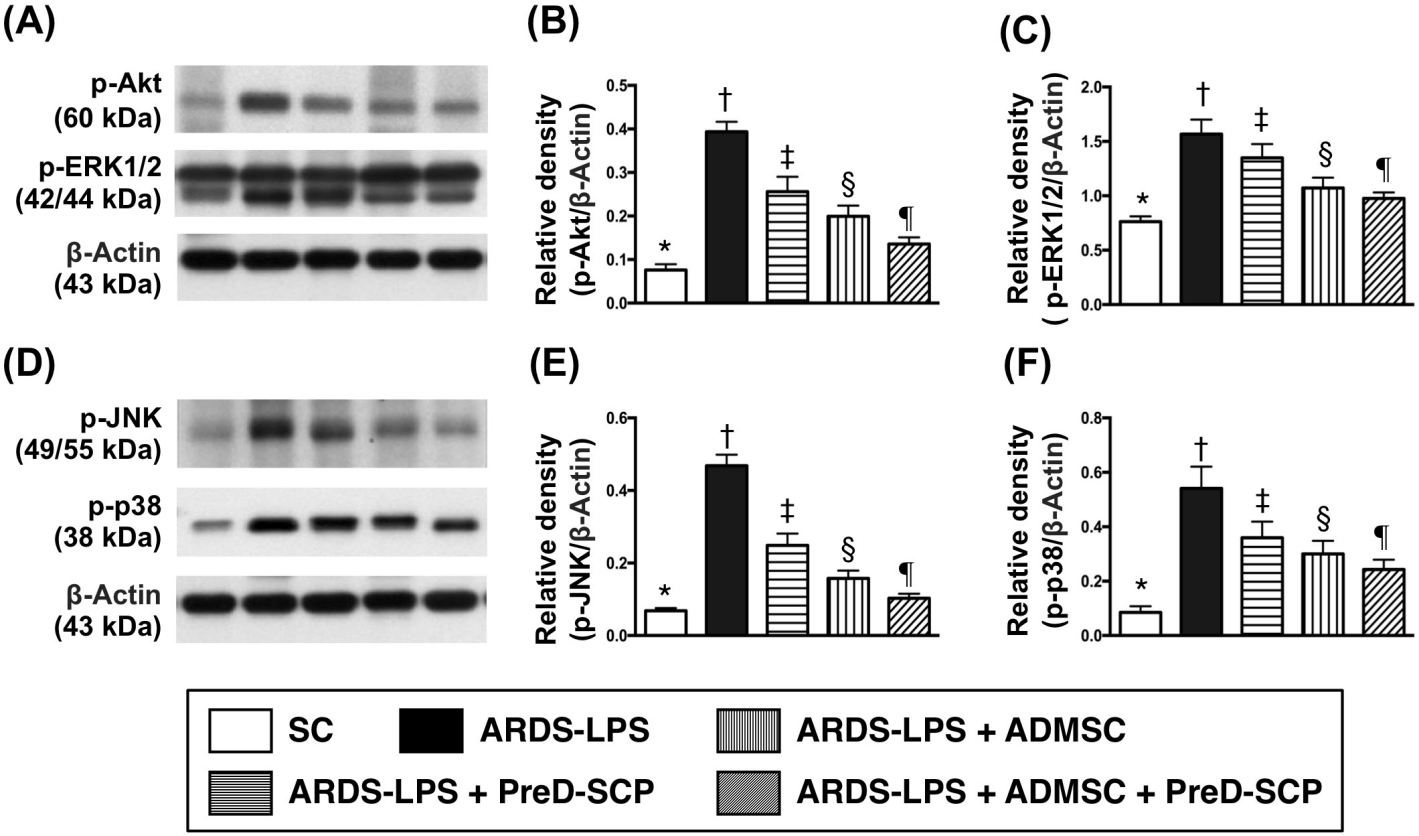
MAPKs and Akt signalings by day 5 after ARDS and sepsis induction Protein levels with different ß-actin as internal control **(A) & (D)** p-Akt **(B)**, p-ERK1/2 **(C)**, p-JNK **(E)**, p-p38 **(F)** * vs. other groups with different symbols (†, ‡, §, ¶), p<0.0001. N=8 for each group. (*, †, ‡, §, ¶) p<0.05. SC, sham control; ARDS-LPS, acute respiratory distress syndrome-lipopolysaccharide; PreD-SCP, preactivated and disaggregated shape-changed platelet; ADMSC, adipose-derived mesenchymal stem cell.

Levels of interleukin (IL)-4, IL-10, and IL-13, three anti-inflammation markers, progressively increased from groups 1 to 5 (Figure 5). This indicated an intrinsically protective response to inflammatory stimulation in the lung. Phosphorylated (p)-ERK1/2, p-JNK, p-p38, three indicators of MAPK family signaling, and p-Akt, an indicator of cell proliferation, differentiation and apoptosis, exhibited the following pattern with respect to measured protein levels: group 2>3>4>5>1 (Figure 6).

### Inflammation, DNA damage, endothelium, and antioxidants in lung parenchyma

**Figure 7:**
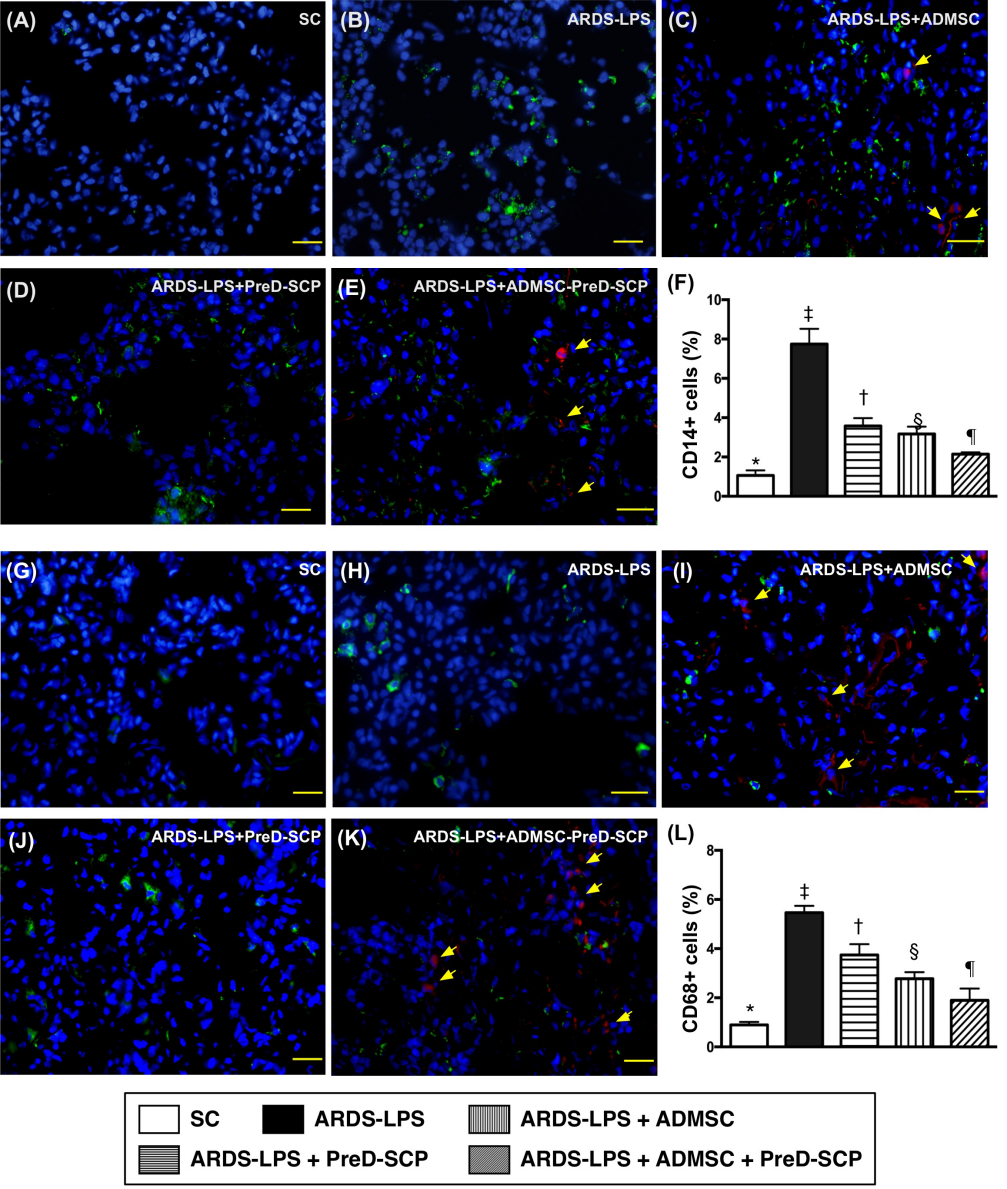
Inflammatory cell infiltration in lung parenchyma 5 d after ARDS and sepsis induction IF microscopy (400x) of CD14+ cells (green color) in lung parenchyma **(A–E)**. Number of CD14+ cells **(F)** * vs. other groups with different symbols (†, ‡, §, ¶), p<0.0001. IF microscopy (400x) of CD68+ cells (green color) in lung parenchyma **(G–K)** Number of CD68+ cells **(L)** * vs. other groups with different symbols (†, ‡, §, ¶), p<0.0001. Blue indicates DAPI stain for nuclei. Red with DAPI-stained nucleus (yellow arrows) in **(C)**, **(E)**, **(I)**, and **(K)** indicate dye-labeled ADMSCs. Scale bars in right lower corner represent 20μm. N=8 for each group. (*, †, ‡, §, ¶) p<0.05. SC, sham control; ARDS-LPS, acute respiratory distress syndrome-lipopolysaccharide; PreD-SCP, preactivated and disaggregated shape-changed platelet; ADMSC, adipose-derived mesenchymal stem cell.

**Figure 8:**
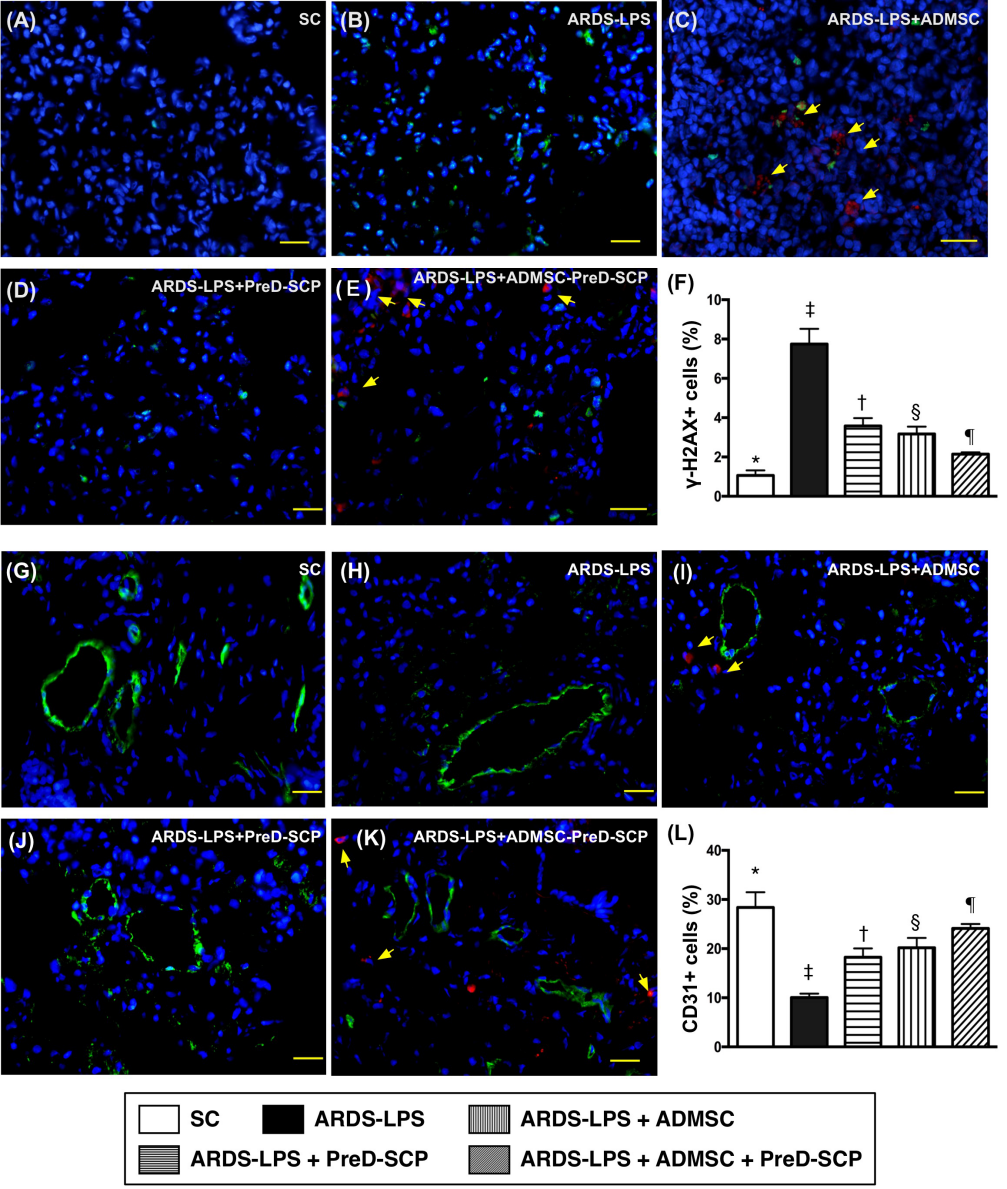
γ-H2AX+ and CD31+ cells in lung parenchyma 5 d after ARDS and sepsis induction IF microscopy (400x) of γ-H2AX+ cells (green color) in lung parenchyma **(A–E)**. Number of γ-H2AX+ cells **(F)** * vs. other groups with different symbols (†, ‡, §, ¶), p<0.0001. IF microscopy (400x) of CD31+ cells (green color) in lung parenchyma **(G–K)**. Number of CD31+ cells **(L)** * vs. other groups with different symbols (†, ‡, §, ¶), p<0.0001. Blue indicates DAPI stain for nuclei. Red with DAPI-stained nucleus (yellow arrows) in **(C)**, **(E)**, **(I)**, and **(K)** indicate dye-labeled ADMSCs. Scale bars in right lower corner represent 20μm. N=8 for each group. (*, †, ‡, §, ¶) p<0.05. SC, sham control; ARDS-LPS, acute respiratory distress syndrome-lipopolysaccharide; PreD-SCP, preactivated and disaggregated shape-changed platelet; ADMSC, adipose-derived mesenchymal stem cell.

**Figure 9:**
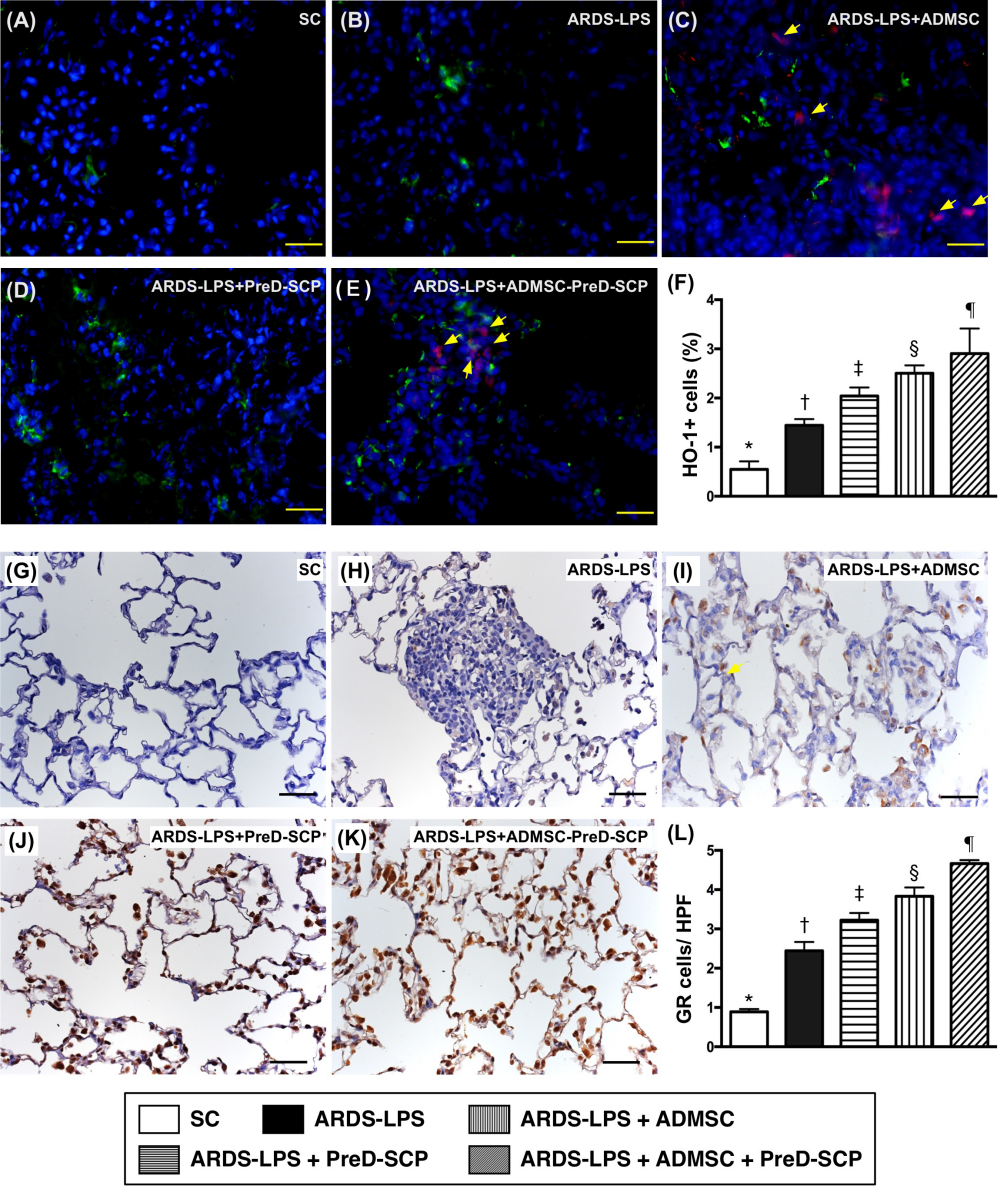
Antioxidants in lung parenchyma 5 d after ARDS and sepsis induction IF microscopy (400x) of heme-oxygenase (HO)-1+ cells (green color) in lung parenchyma **(A–E)**. Blue indicates DAPI stain for nuclei. Red with DAPI-stained nucleus (yellow arrows) in **(C)**, **(E)**, **(I)**, and **(K)** indicate dye-labeled ADMSCs. Scale bars in right lower corner represent 20μm. Number of HO-1+ cells **(F)** * vs. other groups with different symbols (†, ‡, §, ¶), p<0.0001. IHC staining (200x) to identify GR+ cells (gray color) in lung parenchyma **(G–K)**. Scale bars in right lower corner represent 50μm. Number of GR+ cells **(L)** * vs. other groups with different symbols (†, ‡, §, ¶), p<0.0001. N=8 for each group.(*, †, ‡, §, ¶) p<0.05. SC, sham control; ARDS-LPS, acute respiratory distress syndrome-lipopolysaccharide; PreD-SCP, preactivated and disaggregated shape-changed platelet; ADMSC, adipose-derived mesenchymal stem cell.

CD14 and CD68 in lung parenchyma, two indicators of inflammation, and γ-H2AX, an indicator of DNA damage, exhibited the following pattern with respect to measured protein levels: group 2>3>4>5>1 (Figure 7). CD31, an endothelial cell indicator, showed the opposite pattern (Figure 8). HO-1 and GR, two antioxidant indices, increased progressively from groups 1 to 5, suggesting an intrinsic response to ARDS-sepsis stimulation (Figure 9).

## DISCUSSION

This study assessed the benefits of combined ADMSC/PreD-SCP therapy in protecting the lung against ARDS-sepsis injury. In agreement with our previous study [26], we observed lung architectural damage (as measured by lung injury score and crowding score) and impaired functionality (reduced SaO_2_) in rats with ARDS complicated by sepsis, which elicited a rigorous inflammatory response and oxidative stress. MAPK family and Akt signaling pathways were upregulated, which further perpetuated DNA/mitochondrial damage and cell apoptosis. These molecular-cellular perturbations were inhibited by ADMSC/PreD-SCP treatment.

Our recent study showed that PreD-SCP treatment preserved the anatomical and functional integrities of the lung in an ARDS animal model complicated by sepsis [26]. Moreover, we previously demonstrated that ADMSC therapy protected the lung from ischemia-reperfusion [20, 21] and ARDS-induced [27] injuries by reducing inflammation and oxidative stress. The present study found that ADMSC/PreD-SCP combined therapy was superior to either therapy alone for protecting the lung from ARDS complicated by sepsis. Thus, the results of our present study reinforce our previous findings [20, 21, 26, 27] and highlight a novel therapeutic regimen that may be efficacious in critical patients who are refractory to conventional therapy.

Inflammation and oxidative stress resulting from inflammatory cell recruitment and the generation of pro-inflammatory cytokines are fundamental contributors to lung injury in the setting of ARDS/sepsis syndrome [5, 12, 17–21]. We found that the inflammation indicators, NF-κB, MMP-9, RANTES, and TNF-α, along with oxidative stress and mitochondrial damage, were upregulated by ARDS with sepsis in animals. These molecular-cellular events were also identified in our recent study [26]. These findings may partly explain the observed damage to lung architecture and function, as well as enhanced apoptosis and DNA damage in animals with ARDS complicated by sepsis. Importantly, these perturbations were suppressed by ADMSC or PreD-SCP treatment, and were further suppressed by the treatments combined. Anti-inflammatory, anti-oxidant, and anti-apoptotic biomarkers were upregulated after either ADMSC or PreD-SCP treatment alone, and were further increased following combined therapy. These findings demonstrate marked suppression of inflammation and oxidative stress in animals treated with ADMSC and PreD-SCP together, and may partly explain the observed preservation of pulmonary circulation (as measured by CD31 expression and numbers of small vessels), lung architecture, and function in these animals.

The MAPK and Akt pathways direct cellular responses to a diverse range of stimuli, such as mitogens and proinflammatory cytokines. They also regulate various cell functions, including proliferation, gene expression, mitosis, cell differentiation, cell survival, apoptosis and autophagy. Our previous work showed that the MAPK family and Akt were upregulated during myocardial ischemia/infarction, associated increased inflammation with MAPK signaling activation [28], and demonstrated that MAPK signaling promoted pro-inflammatory cytokine upregulation [29]. The present study found that MAPK and Akt signaling, as well as collagen deposition in lung parenchyma, were upregulated in animals with ARDS-sepsis and were suppressed after administration of ADMSC/PreD-SCP.

**Figure 10:**
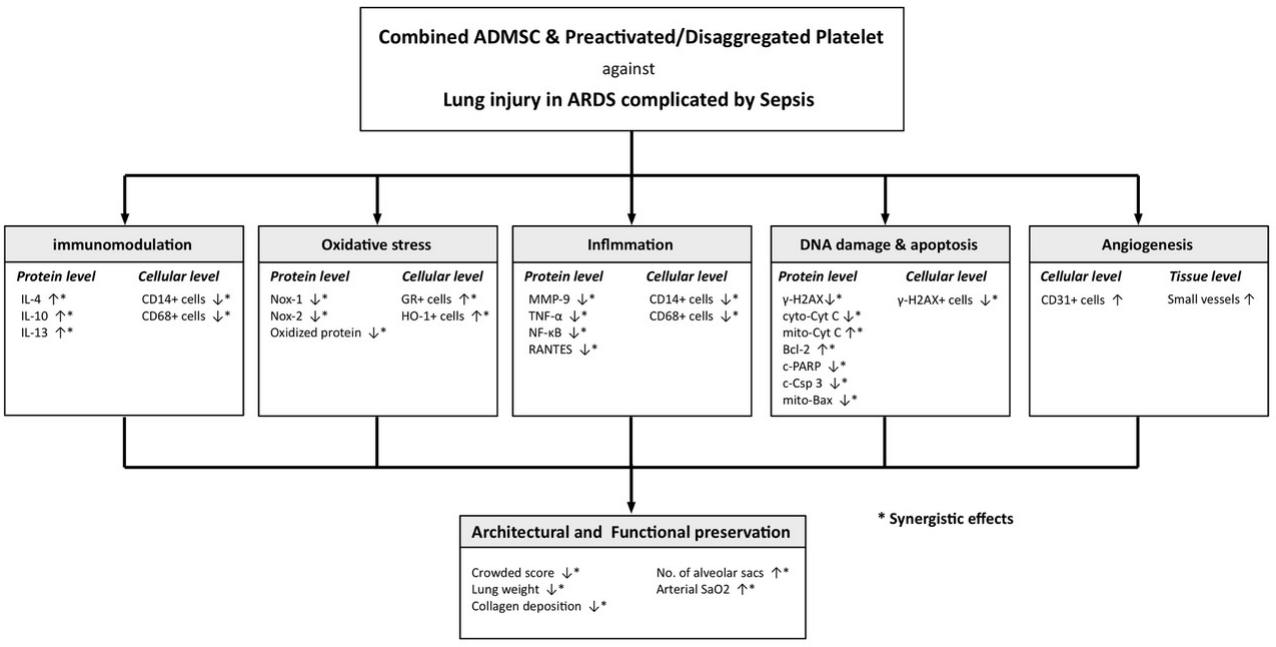
Proposed mechanisms underlying the positive therapeutic effects of combined ADMSC/PreD-SCP on ARDS and sepsis syndrome ARDS, acute respiratory distress syndrome; ADMSC, adipose derived mesenchymal stem cell; IL, interleukin; PARP, poly (ADP-ribose) polymerase (PARP); MMP, matrix metalloproteinase; TNF-α, tumor necrosis factor; NF-κB, nuclear factor; cyto-cyt C, cytosolic cytochrome C; mito-cyt C, mitochondrial cytochrome C.

This study had several limitations. First, the exact mechanisms underlying the observed preservation of both pulmonary architecture and function after ADMS-PreD-SCP treatment in the setting of ARDS complicated with sepsis are still unclear. Additionally, the types of lung cells affected by adoptive cell infusion were not investigated. The proposed mechanisms in the present rat model are summarized in Figure 10. Second, infused ADMSCs that were found to differentiate into other cell types could have been due to the length of time chosen between cell administration and lung specimen harvest (only 72 h). Third, since the study period was only 5 d, animal survival rate and the long-term impact of disease on lung injury are still uncertain.

In conclusion, our results suggest that the mechanisms involved in ARDS complicated by sepsis are likely multi-faceted. Therapeutic intervention reduced inflammation, fibrosis, apoptosis, and oxidative stress, downregulated MAPK/Akt signaling, and upregulated anti-oxidant and anti-inflammatory cytokines. The present study demonstrated that combined ADMSC/PreD-SCP treatment was superior to either therapy alone in reducing ARDS-sepsis-induced acute lung injury and preserving lung function in a rat model.

## MATERIALS AND METHODS

### Ethics statement

All animal experimental procedures were approved by the Institute of Animal Care and Use Committee at Kaohsiung Chang Gung Memorial Hospital (Affidavit of Approval of Animal Use Protocol No. 2013062803) and performed in accordance with the Guide for the Care and Use of Laboratory Animals [The Eighth Edition of the Guide for the Care and Use of Laboratory Animals (NRC 2011)]. Animals were housed in an Association for Assessment and Accreditation of Laboratory Animal Care International (AAALAC)-approved animal facility in our hospital with controlled temperature (24°C) and light cycle (12/12).

### Animal grouping and treatment strategy

Pathogen-free, adult male Sprague-Dawley (SD) rats (n=40) weighing 325-350 g (Charles River Technology, BioLASCO Taiwan Co. Ltd., Taiwan) were randomly divided into five groups: group 1 [normal controls (NC) treated with 1.0 mL normal saline intra-peritoneal (i.p.) injection], group 2 [ARDS + lipopolysaccharide (LPS 1.5 mg/kg, mimicked sepsis syndrome), i.e., ARDS-LPS], group 3 [ARDS-LPS + ADMSC (1.2x10^6^ cells) by intravenous administration], group 4 [ARDS-LPS + PreD-SCP (3.0x10^8^) by intravenous administration], and group 5 (ARDS-LPS + ADMSC + PreD-SCP). The ADMSC and PreD-SCP therapies were performed 24 h after successful ARDS induction.

### Isolation and labeling of adipose-derived mesenchymal stem cells

By d 14, prior to ARDS-sepsis induction, adipose tissue was isolated, as described previously, from groups 3 and 5 animals for culturing ADMSCs [30–32]. Briefly, adipose tissue surrounding the epididymis was carefully dissected, excised and prepared. 200–300 μL of sterile saline was added for every 0.5 g of adipose tissue to prevent dehydration. The tissue was cut into <1 mm^3^-sized pieces using sharp, sterile surgical scissors. Sterile saline (37°C) was added to homogenized adipose tissue at a ratio of 3:1 (saline: adipose tissue), followed by stock collagenase solution to a final concentration of 0.5 units/mL. An aliquot of cell suspension was then removed for cell culture in Dulbecco’s modified Eagle's medium (DMEM)-low glucose medium containing 10% FBS for 14 d. Approximately 2.0–3.0x10^6^ ADMSCs were obtained from each rat.

CellTracker™ Orange CMRA Dye (ThermoFischer Scientific, Taiwan/Molecular Probes, Inc.) (0.5 mM) was added to culture medium to label ADMSCs (1.0x10^6^ cells/batch) 30 min before ADMSC administration.

### An animal model of ARDS complicated by sepsis

An ARDS experimental model was created when pure oxygen (100% O_2_) was continuously administered to rats (from groups 2 to 5) for 48 h [27]. Additionally, to mimic the clinical setting of ARDS complicated by sepsis, LPS (1.5 mg/kg i.p. for each rat) was administered to groups 2 and 5, 24 h after ARDS induction. LPS was administered to animals for sepsis induction as previously described, with minimal modification [26, 33].

### Preparation of non-coagulated pre-activated shape-changed platelets

PreD-SCPs were prepared as previously described [26]. Briefly, 10 additional male adults SD rats were used as blood donors. Under general anesthesia with 2% inhalational isoflurane, 6 mL of blood was obtained through cardiac puncture from each rat. Blood samples were gently transferred to 15 mL centrifuge tubes containing 1 mL of acid citrate dextrose (ACD) buffer (sodium citrate tribasic dihydrate 85 mM, citric acid monohydrate 71mM, dextrose anhydrous 0.1M) at 37°C. A diluting solution containing ACD and normal saline at a volumetric ratio of 1:6 was prepared. Platelet purity and activity were determined by flow cytometry with CD61 (BD Bioscience, Franklin Lakes, NJ, USA) and CD62P (AbD Serotec, Raleigh, NC, USA), respectively.

### Arterial oxygen saturation determination

To investigate the therapeutic effect of ADMSC-Pre-SCP treatment on arterial oxygen saturation (SaO_2_), arterial blood was sampled from the carotid artery for blood gas analysis. Following arterial blood sampling, rats were euthanized and lungs harvested. Lung specimens were prepared for morphometric analyses as previously described [27, 33, 34]. Briefly, the left lung was inflated at a constant airway pressure of 15–20 mmHg and fixed with OCT (Tissue-Tek, Sakura, Netherlands) for immunohistochemical (IHC) staining. The right lung was cut into pieces that were either fixed in 4% paraformaldehyde/0.1% glutaradehyde PBS solution before being embedded in paraffin blocks for hematoxylin and eosin (H&E) staining, or stored at -80°C for protein and mRNA analyses.

### Histological measurement of lung injury

Lung injury score assessments were performed as described previously [27, 33, 34]. Lung specimens were sectioned at 5 μm for light microscopy. H&E staining was performed to estimate the number of alveolar sacs in a blinded fashion. Three lung sections from each rat were analyzed; three randomly selected high-power fields (HPFs; 100x) were examined in each section, and the mean number was determined. The extent of crowded area, which was defined as regions of thickened septa in lung parenchyma associated with partial or complete collapse of alveoli on H&E-stained sections, was also assessed in a blinded fashion. The following scoring system [19, 24, 30] was adopted: 0, no detectable crowded area in a given HPF; 1, <15% crowded area; 2, 15–25%; 3, 25–50%; 4, 50–75%; 5, >75%.

### IHC and immunofluorescent staining

Immunofluorescent (IF) staining was performed as described previously [27, 31, 34]. For IHC and IF staining, rehydrated paraffin sections were treated with 3% H_2_O_2_ for 30 min, then incubated with Immuno-Block reagent (BioSB, Santa Barbara, CA, USA) for 30 min at room temperature. Sections were incubated with primary antibodies against CD68 (1:100, Abcam, Cambridge, MA, USA), CD14 (1:300, BioSS, Woburn, MA, USA), γ-H2AX (1:500, Abcam, Cambridge, MA, USA), CD31 (1:100, AbD serotec), heme-oxygenase (HO)-1 (1:250, Abcam, Cambridge, MA, USA), NAD(P)H quinone oxidoreductase 1 (1:50, Novus Biologicals, Littleton, CO, USA) and glutathione reductase (GR) (1:300, Abcam, Cambridge, MA, USA), while sections incubated with irrelevant antibodies (i.e., p53, CD11, CXCR4,…etc.) served as controls. Three lung sections were analyzed in each rat. For quantification, three randomly selected HPFs (200x or 400x for both IHC and IF studies) were analyzed in each section, and means were calculated. A blinded IHC-based scoring system was adopted for semi-quantitative analysis of GR as a percentage of positive cells [Scores for GR staining: 0 = no stain; 1= <15%; 2 = 15–25%; 3 = 25–50%; 4 = 50–75%; 5= >75%/per high-power filed (200 x)].

To analyze collagen deposition, three lung paraffin sections (4 μm) were stained with picro-Sirius red (1% Sirius red in saturated picric acid solution) for 1 h at room temperature using standard methods. Sections were then washed twice with 0.5% acetic acid. The water was physically removed from the slides by vigorous shaking. After dehydration in 100% ethanol three times, sections were cleaned with xylene and mounted in a resinous medium. Ten low power fields (10x) were used to identify Sirius red-positive areas on each section. Image-pro plus 6.1 software (Media Cybernetics, Bethesda, MD, USA) was used to calculate the total cross-sectional area of the lung and the total Sirius red-positive staining area.

### Vessel density in lung parenchyma

IHC staining of blood vessels was performed with α-SMA (1:400) primary antibody at room temperature for 1 h, followed by three washes with PBS. Ten min after addition of the anti-mouse HRP-conjugated secondary antibody, tissue sections were washed three times with PBS. 3,3’ diaminobenzidine (DAB) (0.7 gm/tablet) (Sigma, St. Louis, Mo, USA) was then added for 1 min, followed by three washes with PBS. Finally, hematoxylin was added for 1 min as a counter-stain for nuclei, followed by three washes with PBS. Three quadriceps sections were analyzed in each rat. For quantification, three randomly selected HPFs (x100) were analyzed in each section, and the mean was calculated.

### Western blot analysis of lung parenchyma

Western blot analyses were performed as previously described [27, 31, 34]. Briefly, equal amounts (50 μg) of protein extracts were separated by SDS-PAGE using acrylamide gradients. After electrophoresis, separated proteins were transferred electrophoretically to a polyvinylidene difluoride (PVDF) membrane (Amersham Biosciences, Amersham, UK). Nonspecific sites were blocked by membrane incubation in blocking buffer [5% nonfat dry milk in TBS containing 0.05% Tween 20 (T-TBS)] overnight. Membranes were incubated with the indicated primary antibodies [mitochondrial Bax (1:1000, Abcam, Cambridge, MA, USA), cleaved PARP (1:1000, Cell Signaling, Danvers, MA, USA), cleaved caspase 3 (1:1000, Cell Signaling, Danvers, MA, USA), Bcl-2 (1:200, Abcam, Cambridge, MA, USA), MMP-9 (1:3000, Abcam, Cambridge, MA, USA), TNF-α (1:1000, Cell Signaling, Danvers, MA, USA), NF-κB (1:600, Abcam, Cambridge, MA, USA), RANTES (1:1000, Cell signaling, Danvers, MA, USA), IL-4 (1:500, Abcam, Cambridge, MA, USA), IL-10 (1:1000, Abcam, Cambridge, MA, USA), IL-13 (1:1000, R&D Systems, Minneapolis, MN, USA), cytosolic cytochrome C (1:1000, BD, San Jose, CA, USA), mitochondrial cytochrome C (1:1000, BD, San Jose, CA, USA), γ-H2AX (1:1000, Cell signaling, Danvers, MA, USA), p-JNK (1:1000, Abcam, Cambridge, MA, USA), p-p38 (1:1000, Abcam, Cambridge, MA, USA), p-ERK1/2 (1:1000, Calbiochem, San Diego, CA, USA), p-Akt (1:1000, Cell signaling, Danvers, MA, USA), NOX-1 (1:2000, Sigma, St. Louis, MO, USA), NOX-2 (1:500, Sigma, St. Louis, MO, USA), and actin (1: 10000, Chemicon, San Diego, CA)] for 1 h at room temperature. Membranes were then incubated with secondary antibody, HRP-conjugated anti-rabbit immunoglobulin IgG (1:2000, Cell Signaling, Danvers, MA, USA), for 1 h at room temperature. The washing procedure was repeated eight times within 1 h. Immunoreactive bands were visualized by enhanced chemiluminescence (ECL; Amersham Biosciences, Amersham, UK) and exposed to Biomax L film (Kodak, Rochester, NY, USA). For quantification, ECL signals were digitized using Labwork software (UVP, Waltham, MA, USA).

### Oxidative stress reaction in lung parenchyma

Oxidative stress-related protein analysis protocols were described previously [27, 31, 34]. The Oxyblot Oxidized Protein Detection Kit was purchased from Chemicon. DNPH derivatization was carried out with 6 μg of protein for 15 min according to the manufacturer’s instructions. One-dimensional electrophoresis was carried out on a 12% SDS/polyacrylamide gel after DNPH derivatization. The protein standard, containing 1-3 dinitrophenylhydrazone (DNP) residues, was loaded on each gel to serve as an internal control for each steps of the Oxyblot. Proteins were transferred to nitrocellulose membranes, which were then incubated with primary antibody (anti-DNP 1: 150) for 2 h, followed by secondary antibody (1:300) for 1 h at room temperature. The washing procedure was repeated eight times within 40 min. Immunoreactive bands were visualized by enhanced chemiluminescence (Amersham Biosciences) and exposed to Biomax L film (Kodak). For quantification, ECL signals were digitized using Labwork software (UVP).

### Statistical analysis

Quantitative data are expressed as means ± SD. Statistical analysis was performed using ANOVA followed by Bonferroni multiple-comparison post hoc test. SAS statistical software for Windows version 8.2 (SAS institute, Cary, NC, USA) was utilized. P<0.05 was considered statistically significant.
